# Applying cosmetic oil with added aromatic compounds improves tactile sensitivity and skin properties

**DOI:** 10.1038/s41598-023-37361-0

**Published:** 2023-06-29

**Authors:** Léonard Samain-Aupic, Laura Gilbert, Nathalie André, Rochelle Ackerley, Edith Ribot-Ciscar, Jean-Marc Aimonetti

**Affiliations:** 1 Aix-Marseille Univ, CNRS, LNC (Laboratoire de Neurosciences Cognitives - UMR 7291), 3 place Victor Hugo, 13003 Marseille, France; 2 Laboratoires Clarins, 5 rue Ampère, 95300 Pontoise, France

**Keywords:** Neuroscience, Physiology, Psychology, Health care

## Abstract

Tactile sensitivity generally decreases with aging and is associated with impairments in skin properties. Products that hydrate the skin can combat touch deficits and aromatic compounds have been shown to improve skin mechanical properties. Thus, we tested a base cosmetic oil against a perfumed oil, applied to the skin of females aged 40–60 years, on tactile sensitivity and skin properties after repeated application. Tactile detection thresholds were assessed using calibrated monofilaments applied at the index finger, palm, forearm, and cheek. Spatial discrimination on the finger was assessed using pairs of plates with different inter-band spaces. These tests were performed before and after 1 month of base or perfumed oil use. We found that tactile detection thresholds and spatial discrimination improved only in perfumed oil group. A complementary immunohistological study using human skin was conducted to estimate the expression of olfactory receptor OR2A4 and elastic fiber length. Further, the expression of OR2A4 intensity and the length of elastic fibers increased significantly with oil application, where larger effects were seen with the perfumed oil. We conclude that the application of a perfumed oil may be of additional benefit and could repair, and even prevent, tactile decline with aging by ameliorating skin condition.

## Introduction

Tactile sensitivity, our ability to process mechanical events on the body, generally declines with age^1^. Aging is strongly associated with a decrease in the number of peripheral mechanoreceptors and/or degenerative changes in their fibers^2,3^. Such a deterioration of the encoding and transmission of tactile information can have a major impact of touch. Further, central factors such as loss of white matter and reduced blood flow in the somatosensory areas can diminish tactile processing^4,5^. However, skin physical properties are also impaired with age, where the synthesis of collagen and elastin decreases, which impedes skin repair^6^. Lower skin elasticity also changes the transmission of mechanical events, affecting the encoding of touch^7^. Protection of the skin is important to combat these physical and biomechanical effects of aging, thus any product that improves skin mechanical properties may impact tactile sensitivity. This would also be a relatively simple and cost-effective route to sustain and improve touch. Previous work has demonstrated that touch detection thresholds and spatial discrimination improve immediately after applying a moisturizing cream, likely due to increased skin hydration^7–9^. As well short-term benefits of skin cream application, tactile spatial discrimination has been shown to be significantly improved after 1 month of cream application^10^.

Damage to the external layers of the skin can produce dryness, but also far more serious effects, such as wounds and irritation. These create problems for the microanatomy of the skin (e.g. cellular level) and its whole integrity at a more macrolevel (e.g. layer damage, decline in afferent function). Changes in the lipid profile of the skin can be especially damaging, where water loss can lead to exposure to irritants and risk of infection^11^. The principal protective layer of the skin, the epidermis, is mostly composed of keratinocytes, thus all mechanical stimuli first contact this layer. Keratinocytes play a number of important roles in the skin. Mechanoreceptors and their afferent fibers are embedded in the skin below this layer, where the keratinocytes provide a supportive role, but may also help transduce mechanical stimuli to afferents^12–14^. Further, keratinocytes contain the highest concentration of antioxidant enzymes of the skin and actively contribute to skin repair^6^. Human keratinocytes have been found to express the olfactory receptor OR2A4^15^. Its activation by odorant molecules, such as sandalwood, has been shown to increase keratinocyte proliferation and migration during wound healing^15,16^.

Considering the beneficial effects of long-term skin hydration on touch and that olfactory receptor activation can improve skin mechanical properties, we compared tactile sensitivity before and after 1 month of skin oil application in two groups, comparing a ‘base oil’ (hazelnut seed) with a ‘perfumed oil’ (same base, with odorant molecules including *Pogostemon cablin* (patchouli) oil). We predicted that 1 month of oil application would improve skin properties and, consequently, tactile measures in both groups. In the perfumed oil group, we additionally hypothesized that the odorant molecules would activate skin olfactory receptors, improving skin properties, which would further improve tactile sensitivity. Tactile sensitivity was estimated in terms of detection threshold on the index finger, palm, forearm, and cheek, as well as the spatial discrimination of the index, in two groups of middle-age women. In addition, skin effects of both oils were compared in ex-vivo immunohistochemical experiments.

## Results

### Touch tests: tactile detection threshold

**Figure 1:**
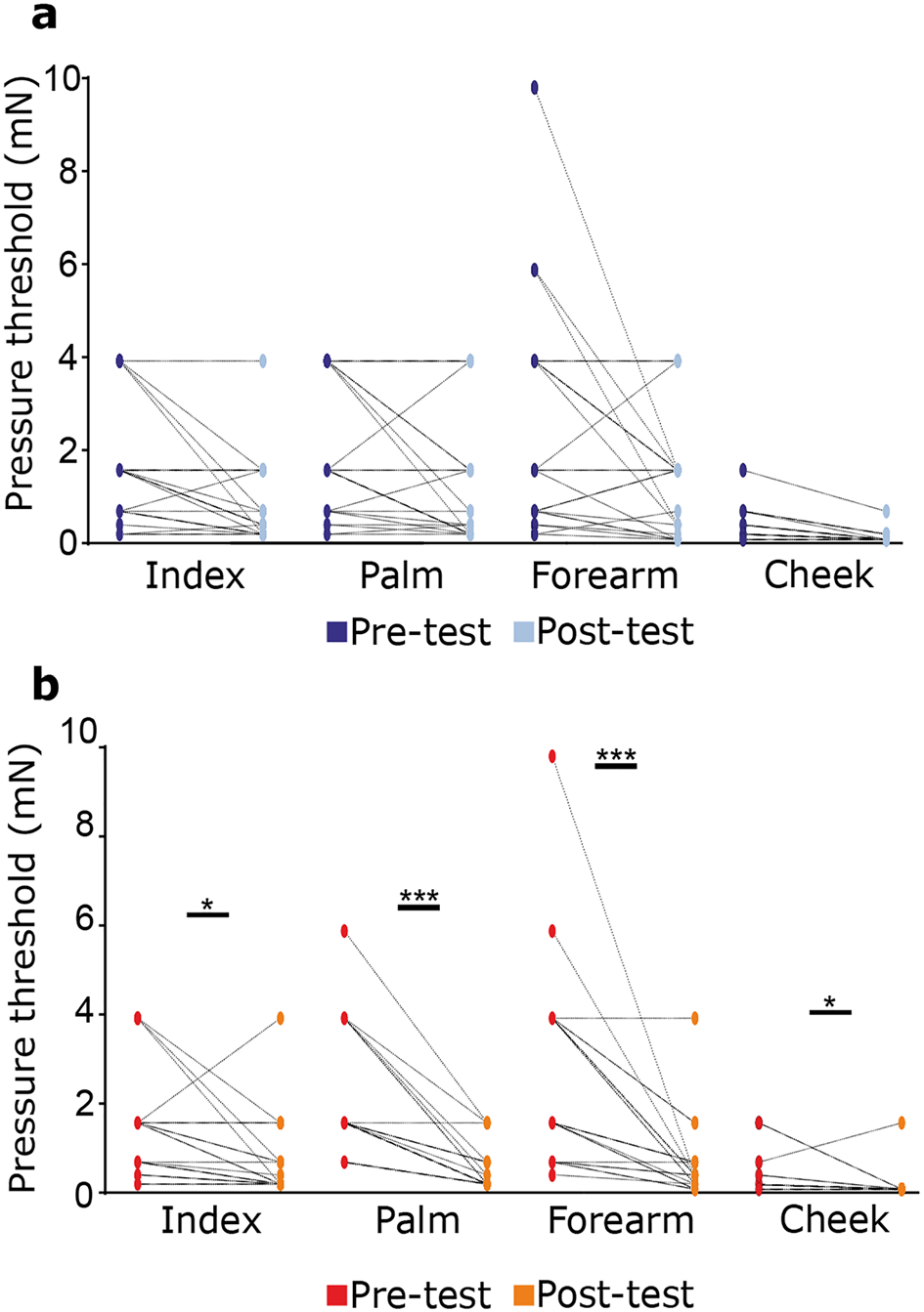
Tactile detection thresholds before and after oil application over different body sites, for the base and perfumed oils. Tactile detection thresholds at 95% before and after cosmetic oil application in the (**a**) base oil group and (**b**) perfumed oil group. Individual thresholds are shown. *p < 0.05, **p < 0.01, ***p < 0.001.

In a baseline pre-test, before oil application, no differences were observed in the tactile detection threshold using calibrated monofilaments, between the two participant groups for each skin site tested (all Dunn’s multiple comparison tests p > 0.999). After 1 month of oil application, the tactile detection threshold remained unchanged in the base oil group (Fig. 1a; FDR-corrected post-hoc comparisons: index p = 0.074, mean pre = 1.6 mN ± 0.3, post = 0.8 mN ± 0.2; palm p = 0.101, mean pre = 1.9 mN ± 0.3, post = 1.3 ± 0.3; forearm p = 0.074, mean pre = 2.5 mN ± 0.5, post = 1.3 mN ± 0.3; cheek p = 0.074, mean pre = 0.4 mN ± 0.1, post = 0.1 mN ± 0.03). By contrast, in the perfumed oil group, tactile detection thresholds decreased for all sites (Fig. 1b; FDR-corrected post-hoc comparisons index p = 0.047, mean pre = 1.3 mN ± 0.3, post = 0.7 mN ± 0.19; palm p < 0.001, mean pre = 2 mN ± 0.3, post = 0.5 mN ± 0.1; forearm p < 0.001, mean pre = 2.7 mN ± 0.5, post = 0.6 mN ± 0.2; cheek p = 0.047, mean pre = 0.3 mN ± 0.1, post = 0.2 mN ± 0.1).

### Touch tests: tactile spatial discrimination

**Figure 2:**
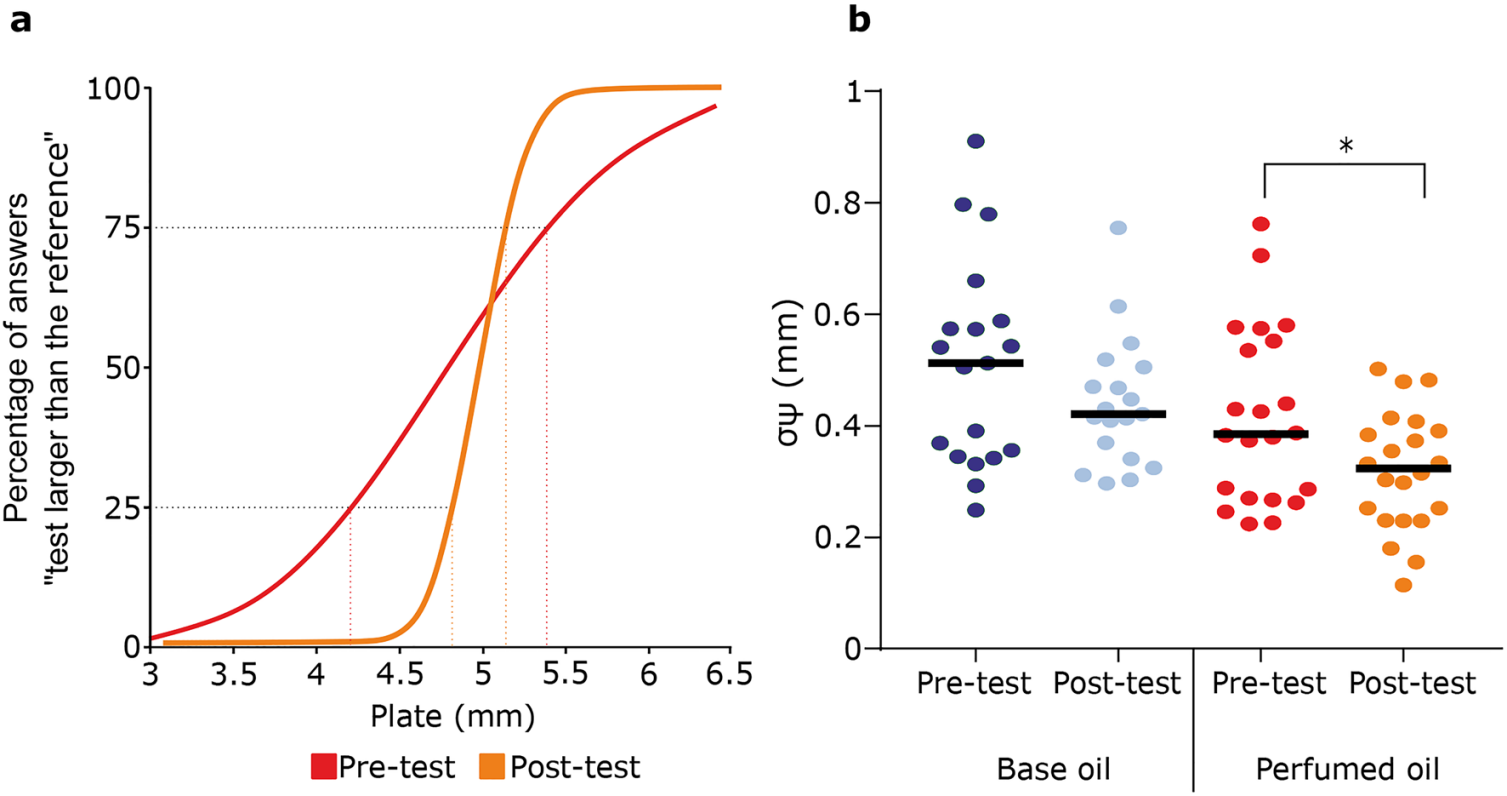
Tactile spatial discrimination at the index finger before and after oil application, for the base and perfumed oils. (**a**) Individual psychophysical curve for tactile spatial discrimination before perfumed oil application and after 1 month of oil application. (**b**) Group spatial discrimination difference thresholds (σψ) in the base and perfumed oil groups before and after 1 month of oil application, for the base and perfumed oils. Individual participants are shown, with the mean indicated as the black lines. *p < 0.05.

Figure 2a illustrates a typical psychophysical curve of a participant’s tactile spatial discrimination before and after perfumed oil application. Here, the threshold for discrimination was lower after oil application (before 580 µm, after 154 µm). No significant difference in tactile spatial discrimination threshold was found between the groups before oil application (t = 1.71, p = 0.096). After 1 month of oil application, the perfumed group had a significantly improved tactile spatial discrimination threshold (Fig. 2b; t = 2.37, p = 0.027; mean before 417 µm ± 34, after 318 µm ± 23). In the base oil group, the spatial discrimination threshold showed a tendency to decrease from 508 µm ± 42 to 440 µm ± 27, but the higher pre-test variability meant that this decrease was not significant (Fig. 2b; t = 1.86, p = 0.079).

### Immunohistochemistry: OR2A4 receptor intensity

**Figure 3:**
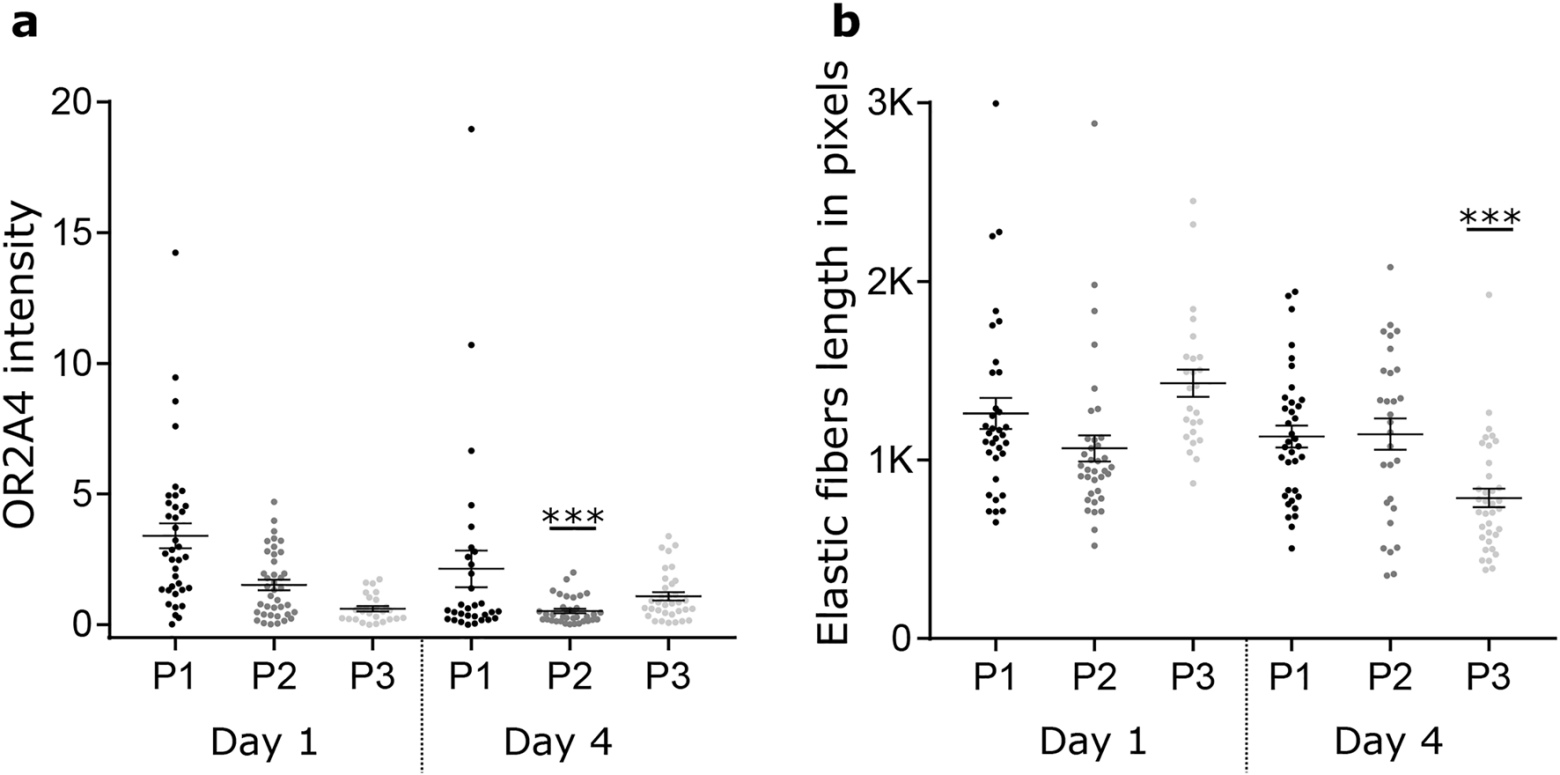
Quantification of OR2A4 intensity and elastic fiber length in the three plasties in the control condition. (**a**) OR2A4 immunofluorescence intensity around the epidermis keratinocytes reported to DAPI area of the keratinocytes by the three plasties (P1–P3) at days 1 and 4 in the control condition. (**b**) Same data for the three plasties in terms of elastic fiber length.

Three human skin explant plasties were used to assess immunofluorescent intensity of OR2A4 under control (no oil), base oil, and perfumed oil conditions, which were assessed at day 1 and day 4. In the control skin preparations, the only significant change was a decrease in OR2A4 intensity in plasty 2 after 4 days (mean OR2A4 intensity day 1 = 1.5 ± 0.2; day 4 = 0.5 ± 0.1, p < 0.001; Fig. 3a).

**Figure 4:**
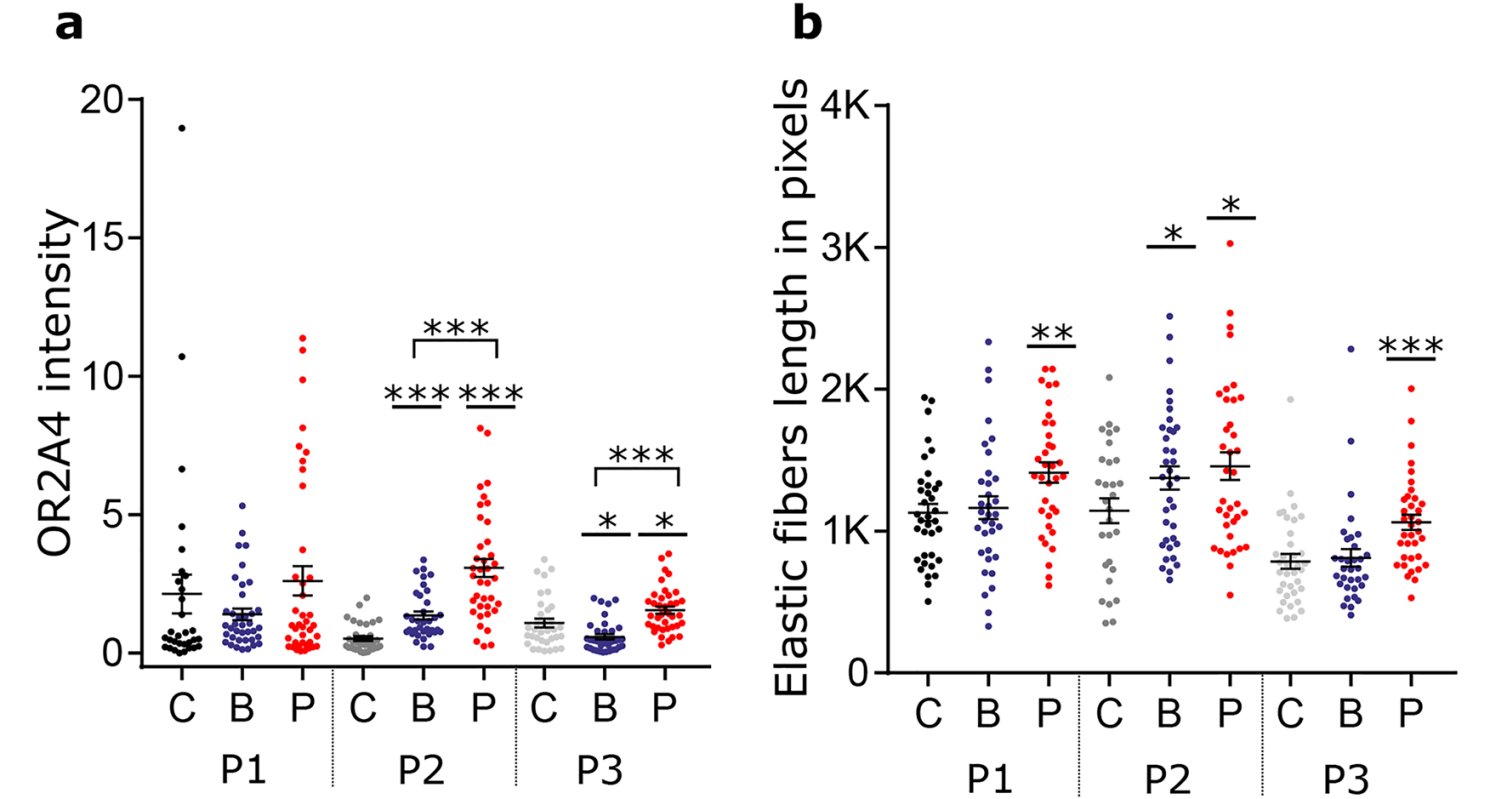
Quantification of OR2A4 intensity and elastic fiber length in the three plasties in all conditions at day 4. (**a**) OR2A4 immunofluorescence intensity around the epidermis keratinocytes reported to DAPI area of the keratinocytes for the three plasties (P1-P3) in all conditions. (**b**) Elastic fiber length expressed in pixels for the three plasties in all conditions. *C* control, *B* base oil, *P* perfumed oil.

**Figure 5:**
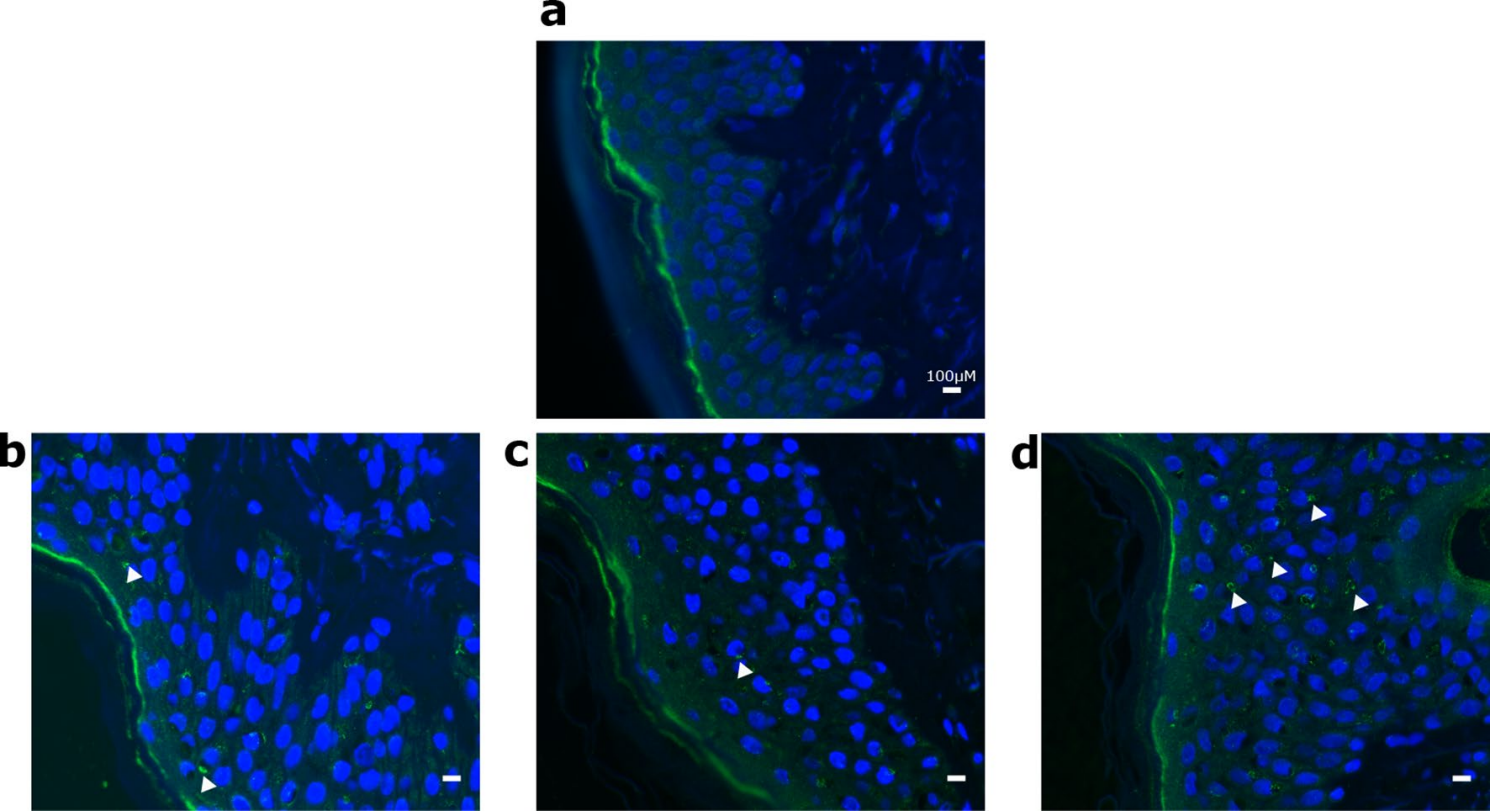
Expression of immunoreactive olfactory receptor OR2A4. The expression of OR2A4 is seen in the green ring shapes around keratinocytes, as pointed to with white triangles. Nuclear immunostaining with DAPI (blue) in the epidermis is also shown. These data were collected from plasty 3 at (**a**) day 1 baseline, (**b**) day 4 control, (**c**) day 4 base oil, (**d**) day 4 perfumed oil.

For plasties treated with base oil application, OR2A4 immunofluorescent intensity increased in one plasty after 4 days (plasty 2 p < 0.001, mean OR2A4 intensity: control = 0.5 ± 0.1, base oil = 1.4 ± 0.1) and decreased for one plasty (plasty 3 p = 0.045, control = 1.1 ± 0.2, base oil = 0.6 ± 0.1). It significantly increased with the perfumed oil in two plasties (plasty 2 p < 0.001, mean = 3.1 ± 0.3; plasty 3 p = 0.014, mean = 1.5 ± 0.1). Further, the intensity of OR2A4 fluorescence was higher with perfumed oil than with the base oil (plasty 2 p < 0.001; plasty 3 p < 0.001). Data on the OR2A4 expression from all plasties are shown in Fig. 4a and the whole time series of OR2A4 receptor expression for plasty 3 is illustrated in Fig. 5.

### Immunohistochemistry: elastic fiber length

The length of the elastic fibers at day 4 are shown in Fig. 4b. In the control (no oil) condition, elastic fiber length remained unchanged after 4 days in plasties 1 and 2, but a significant decrease was observed in plasty 3 (p < 0.001, mean elastic fiber length in pixels, day 1 = 1429 ± 76, day 4 = 787 ± 52; Fig. 3b). After 4 days, elastic fiber length significantly increased for all the plasties treated with perfumed oil (plasty 1 p = 0.004, mean control = 1131 ± 61, perfumed oil = 1413 ± 72; plasty 2 p = 0.033, mean control = 1145 ± 88, perfumed oil = 1459 ± 97; plasty 3 p < 0.001, mean control = 787 ± 52, perfumed oil = 1062 ± 54), while it increased significantly only for plasty 2 with the base oil (p = 0.016, mean control = 1145 ± 88, base oil = 1375 ± 82).

## Discussion

We investigated the impact of the 1-month application of a base and a perfumed cosmetic oil on tactile sensitivity and compared these with immunohistological experiments on human skin explants over 4 days, from different people undergoing abdominoplasty. For tactile sensitivity, touch detection thresholds decreased only in the group who applied perfumed oil, regardless of the skin area tested. Similarly, tactile spatial discrimination threshold of the finger improved only in the perfumed oil group. The immunohistochemical results showed that after 4 days of culture, the expression of olfactory receptors and the length of elastic fibers were increased after oil application, but these were larger with the perfumed oil.

Tactile detection thresholds significantly decreased after 1 month of oil application over all sites tested only in the perfumed oil group. This occurred over both glabrous skin (finger, palm) and hairy skin (forearm, cheek). A similar tendency was observed in the group using the base oil, but this was not statistically significant. We did not test tactile discrimination at all skin sites, but when tactile spatial discrimination was tested in the active touch paradigm using the glabrous finger skin, improvements were observed only in the perfumed oil group, where the participants gained ~ 100 µm in spatial discrimination acuity, which is close to the gain of 80 µm found previously after 1 month of cosmetic cream application in an older age group (60–75 years^10^). This suggests that cosmetic skin hydration may improve tactile sensitivity as early as 40 years old, contrary to what has been previously reported^8^. Overall, we found similar touch amelioration across heterogeneous skin regions (i.e. between glabrous and hairy skin, different skin thicknesses), leading us to conclude that the effects, likely due to increased structural skin restoration, could be extended across the body. Further explorations of tactile capacity improvements of odorous product application over the skin are warranted, such as other tactile discrimination tests, tactile vibration perception, and tactile pleasantness perception.

The results from testing stomach skin explants, from hairy type skin, showed complementary changes in terms of both OR2A4 intensity and elastic fiber length. The expression of olfactory receptor OR2A4 was significantly greater after 4 days in two out of the three plasties for perfumed oil application and just in one plasty for base oil application. This suggests that although a simple hazelnut oil may be sufficient to promote the expression of olfactory receptors, the addition of aromatic compounds may enhance the process. This demonstrates that the tactile improvements in the other tests may be linked to fundamental skin structure, such as the changes we found in OR2A4 intensity, which has been associated with keratinocytes and their impact on skin health^15,16^. We also evaluated the effects on elastic fiber length, a marker of skin elasticity known to be reduced with aging^17^, and found that oil application enlarged these fibers overall, but the increases were more consistent over time with perfumed oil. Our quantification of elastic length was a semi-automatic method, thus more quantitative investigations (e.g. electron microscopy) are warranted. An increase in elastic fiber length could change the mechanical properties of the skin, and thus, the responses of mechanoreceptors. Although the exact skin used between psychophysical and ex vivo tests was not identical, the explant tests were driven by the availability of human skin. However, both tests were controlled in terms of sex and age range and the abdomen skin is similar to that of the arm, in terms of type and thickness^18,19^.

Among the mechanisms responsible for short term changes in tactile sensitivity after cosmetic product application, skin hydration has been highly implicated^7–9^, and can even change roughness perception^20^. Finger skin elasticity has also been found to influence tactile discrimination^9^. Such biomechanical skin properties, which are often impaired in old age^6^, may contribute to losses in tactile sensitivity and any product that improves these should aid tactile sensitivity. This peripheral mechanism is supported here by the lower tactile detection threshold observed over all skin sites in the group using the perfumed oil.

Among the other possible peripheral mechanisms responsible for the additional tactile improvement, we propose that the activation of the olfactory receptors, discovered in human keratinocytes, may be involved, as they can improve skin mechanical properties^15,16^ and we saw specific improvements when aromatic compounds were included in oil application. These immunological results are in line with recent studies. For example, a rose extract liable to activate skin olfactory receptors has been found to significantly reduce specific markers of skin alteration, in both skin explants and using a clinical approach, after a month of application^21^. Similarly, Tephrosia purpurea extract has been shown to reduce skin changes both with ex-vivo and in-vivo approaches^22^. Finally, extract of *Potentilla erecta* has been found to accelerate wound healing in rats and increase the health of skin, by altering oxidative events^23^. Thus, it seems pertinent to explore how aromatic compounds can aide the structure of the skin, especially as this could be a non-medical, cost-effective route to improve skin well-being.

Our combined psychophysical and immunohistochemical approaches suggest that the regular application of cosmetic oil itself may prevent skin aging impairments, likely through skin hydration, with these effects being greater when aromatic compounds are added. We found more consistent improvements in plasties exposed to perfumed oil, and combined with the improvement in tactile sensitivity, lead us to suggest that a preventive application of perfumed cosmetic oil may sustain tactile sensitivity during aging, which has effects even from middle age. Although intrinsic aging can be slowed, it is inevitable, thus aging from the molecular and cellular levels are difficult to combat^24^. Thus, it is unlikely that the application of skin hydrating products can change the mechanisms of mechanotransduction at the level of PIEZO receptors^25^, nor the have an effect on mechanoreceptor density and innervation, but the degradation of such processes may be reduced by preserving the mechanical structure and properties of the skin.

However, this skin amelioration likely works in tandem with other phenomena, such as peripheral and central neural mechanisms. Skedung et al*.*^9^ reported variability in tactile sensitivity in elderly people, where high and low tactile performers had similar skin mechanical properties, but the density of mechanoreceptors was sustained in high performers. One can also consider the long-term effects of hand use, where specific professional skills may impair or preserve tactile function^26,27^. Further, we show results from a constrained group, only on females, but we postulate that our findings should be generalizable and could be even greater in people with drier skin and with age.

Until now, the cosmetic industry has focused on the prevention of skin aging and the drive for an eternally youthful appearance^28^. We conclude that preserving skin properties, such as hydration and elasticity, may help sustain and even ameliorate somatosensory function. Further, the benefits of odorant molecules for the skin should be explored, especially as they have been already studied for treating burns^15,21,29^. As well as the direct skin benefits of aromatic compounds, the odor of a cosmetic product, combined with its tactile properties, has been shown to increase well-being in different countries^30^, therefore the whole multisensory experience can be capitalized upon for increased benefits.

## Material and methods

### Psychophysical touch sensitivity tests

#### Participants

Experiments were performed on 44 healthy adult women (22 in each group) aged between 40 and 60 years old. The study was approved by an ethical committee (Comité de protection des personnes Est-III) and all participants gave their written informed consent. The investigation was carried out in accordance with the Declaration of Helsinki, except for preregistration in a database. Participants were recruited according to their response to a written survey about cosmetic product use. They had to use a night facial care at least 4 times per week and have normal or dry skin. The exclusion criteria were history of neurological, psychiatric, or dermatological disorder, peripheral neuropathy, or pregnancy. The Edinburgh Handedness Inventory was performed in order to test hand dominance (one participant left-handed). The participants were assigned randomly to either the base oil group (mean age 50.6 ± 4.1 years old) or perfumed oil group (mean age 50.6 ± 5.1 years old) and the study was conducted double-blind. All tests were conducted on the right side of the body, apart from the left-handed person, where the tests were on the left side.

#### Products

Two cosmetic oils were used: a hazelnut seed oil, named throughout as the ‘base oil’, which was used by one group of participants, and the same oil (97.6%) with added odorous compounds (2.4%), named throughout as the ‘perfumed oil’, that was applied by a second group. The ‘perfumed oil’ is a commercial face oil from Clarins (France; Blue Orchid Treatment Oil made from 100% natural plant extracts including *Pogostemon* *cablin* (patchouli) oil).

#### Tactile sensitivity tests

All the tests took place in the same laboratory room and the temperature was controlled at 21 °C. Tests were performed before any application of the oil and after 1-month application of the oil. For 30 days, participants applied oil to their face, on their hands, and on their forearms (5 drops per site) every night before going to bed, without using any other cosmetic product.

#### Tactile detection test

Tactile detection thresholds were measured using 13 calibrated monofilaments (range: 78, 59, 39, 20, 14, 10, 6, 4, 1.6, 0.7, 0.4, 0.2, 0.08 mN) and were applied on the middle of the index fingertip, palm, cheek, and ventral forearm. The order of tested areas was pseudo-randomized. The participant had to respond when they felt being touched by saying “yes”. They closed their eyes and wore noise-canceling headphones (Bose) to avoid both visual and auditory clues. A staircase method was used to establish a detection threshold. The test started using the 39 mN monofilament and the experimenter applied it three times. If there were three correct responses, the monofilament level decreased by two (14 mN). The next monofilament used was the adjacent higher force level (20 mN). The procedure then continued in this step-wise order until an incorrect detection was given (miss). If the participant missed a detection, the adjacent higher force level was tested. This procedure was terminated when two error were made and the monofilament above was noted as the tactile detection threshold^31^.

#### Tactile spatial discrimination

Tactile spatial discrimination was tested on the distal phalanx of the index of the dominant hand using poly-methyl-methacrylate plates with grooved bands^10^. Each band had perceptible depth of 0.07 mm and the 11 plates had inter-band groove spacing, ranging from 3.6 to 6 mm. The plate with medium-spacing of 4.8 mm was the reference. The inter-band spacing of the other test plates varied by a step of 0.2 mm except for the two extreme plates, where the spacing change by a step of 0.4 mm (see^10^). Participants had to explore pairs of plates where the reference plate was compared to a test plate. A familiarization phase was performed to train the participant to explore the plate, using a single vertical movement at about 2 cm/s, which is typical when exploring a surface^32^. The test consisted of a two-alternative forced choice discrimination, where the participant had to determine which plate had the larger inter-band spacing between the reference and a test plate, exploring one at a time. The extreme (easier, furthest from the reference) plates were presented six times and all others 15 times. A total of 132 reference-test plate pairs were presented for each participant in a random order. The responses were expressed and modeled in the percentage of responses when the participant said that the test plate was more spaced than reference plate, and psychophysical curves were gained.

### Skin ex-vivo experiments

Three healthy skin explants were prepared from abdominal plastic surgery from adults between 40–60 years old of skin phototype II according to the Fitzpatrick classification, who gave written informed consent and did not participate in the other part of the study. The first and the second were 42 years old and the third 48 years old. Three skin preparations per plasty were cut with a depth of 18 mm and prepared by removing fatty tissue, placing the samples on a sterile metal grid, and sustaining them throughout the study with a maintenance culture medium (2/3 IMDM medium + 1/3 keratinocyte medium, 10% fetal cow serum), infused via the dermal layer.

Each plasty was sectioned in three to test the application of the base or perfumed oil, and was compared to an untreated control, over 4 days. Each section was further sectioned in three to evaluate OR2A4 receptor expression and elastic fiber length repeatability. Each condition with the base or perfumed oil was thus compared to its untreated control plasty and to the repetition of its condition. Skin sample controls at day 1 were taken to evaluate the skin quality at the beginning of the experiment and constituted the basal state skin (reference skin control) for the different observed parameters (olfactory receptor OR2A4 and elastin) after 4 days. Application of the base or perfumed oil was performed daily, and the culture medium was changed three times a week. Although the explants were kept for at least 8 days, the data obtained on the eighth day timepoint were variable, due to preparation conservation difficulties of living skin cells. However, these data are available on request to the authors. At the end of the different sample times, skin explants were fixed in formalin during 24 h, embedded in paraffin, and 5 µm slices were cut.

For OR2A4 immunohistochemistry, plasty cuts were dewaxed and permeabilized by Triton 0.1% in phosphate buffer solution (PBS), then saturated by bovine serum solution 2% in PBS. Slices were then incubated with rabbit olfactory receptor antibody OR2A4 (NLS3903 Biotechne), 1/50 in bovine serum albumin solution 1% in PBS, for 1 h at room temperature, rinsed three times in PBS and incubated with Alexa Fluor 488 anti-rabbit (A11088 Life technologies) 1/200 and DAPI stain nuclear marker (D9564 Sigma) for 1 h at room temperature, and protected from light. Slices were conserved, protected from light, at 4 °C until fluorescence microscopy.

For elastic fiber immunohistochemistry, cuts were permeabilized, saturated, and then incubated with mouse elastin antibody (Millipore MAB2503) 1/100 in bovine serum albumin solution 1% in PBS, for 1 h at room temperature, rinsed three times in PBS, and incubated with Alexa Fluor 488 anti-mouse (A11001 Life technologies) 1/200 and DAPI stain, nuclear marker (D9564 Sigma) for 1 h at room temperature, and protected from light. Slices were conserved, protected from light, at 4 °C until fluorescence microscopy.

Immunohistochemical acquisitions of OR2A4 and elastin were performed on a Nikon fluorescence microscope. Immunohistochemical analyses were performed on Visilog software by semi-automatic method by determining the fluorescence threshold of OR2A4. The results were expressed in pixels. Results for OR2A4 were expressed by calculating the ratio of immunofluorescence of OR2A4 in the epidermis keratinocytes reported on the DAPI’s area. Elastic fiber length was expressed in fluorescence pixels. This consists in a quantitative immunofluorescence analysis developed in-house at Clarins laboratories that estimates the length of unfragmented elastic fibers in pixels.

### Statistical analysis

For all data, means are given with standard errors of the mean (± SEM). Due to the violation of the normality in Shapiro–Wilk tests, non-parametric statistics were used for the tactile detection test. A Kruskal–Wallis test with Dunn’s multiple comparison correction was performed to compare results directly between the groups. A Friedman ANOVA was used to compare the results in each group, applying a false discovery rate correction^33^ to post-hoc comparisons.

For spatial tactile discrimination, psychophysical curves and the extracted parameters were calculated with Palamedes toolbox^34^ in MATLAB (Mathworks). The difference threshold (σψ) corresponded to the difference between the projection onto the x-axis at 75% and 25% correct responses divided by two (see Fig. 2)^10^. Data for the difference threshold followed a normal distribution, and a paired Student’s *t* test was used to test the effect of the application of the different oils. Z-scores were calculated as Z = (x − µ)/σ, where x corresponds to the individual result, µ to the mean, and σ to the standard deviation. A high Z-score means that the result of the participant was far from the average, and thus considered as outlier. Three participants from the base oil group were excluded from the analyses, as they were deemed unable to do the task because their performance did not reach at least 90% of correct response for the comparison of the easiest pair. According to the z-score calculated, their data were excluded.

The expression of olfactory receptor OR2A4 (DAPI area) and the length of elastic fibers (pixels) were expressed as means ± SEM, with *n* as the number of measurement pictures per skin. Due to the non-normality of the data, Wilcoxon rank tests were performed for the analyses of the OR2A4 expression and elastic fiber length.

## Data Availability

The raw data supporting the conclusions of this manuscript will be made available by the authors, without undue reservation, to any qualified researcher.
